# Knockdown expression of a MYB-related transcription factor gene, *OsMYBS2*, enhances production of recombinant proteins in rice suspension cells

**DOI:** 10.1186/s13007-021-00799-2

**Published:** 2021-09-25

**Authors:** Desyanti Saulina Sinaga, Shin-Lon Ho, Chung-An Lu, Su-May Yu, Li-Fen Huang

**Affiliations:** 1 Graduate School of Biotechnology and Bioengineering, Yuan Ze University, Taoyuan City, 320 Taiwan, ROC; 2 Department of Agronomy, National Chiayi University, Chiayi City, 600 Taiwan, ROC; 3 Department of Life Sciences, National Central University, Taoyuan City, 320 Taiwan, ROC; 4 Institute of Molecular Biology, Academia Sinica, Nankang, Taipei City, 115 Taiwan, ROC

**Keywords:** OsMYBS2, Rice suspension cells, Sugar, *αAmy3* promoter, Recombinant protein, Mouse GM-CSF

## Abstract

**Background:**

Transgenic plant suspension cells show economic potential for the production of valuable bioproducts. The sugar starvation-inducible rice *αAmy3* promoter, together with its signal peptide, is widely applied to produce recombinant proteins in rice suspension cells. The OsMYBS2 transcription factor was shown recently to reduce activation of the *αAmy3* promoter by competing for the binding site of the TA box of the *αAmy3* promoter with the potent OsMYBS1 activator. In this study, rice suspension cells were genetically engineered to silence *OsMYBS2* to enhance the production of recombinant proteins.

**Results:**

The mouse granulocyte–macrophage colony-stimulating factor (mGM-CSF) gene was controlled by the *αAmy3* promoter and expressed in *OsMYBS2*-silenced transgenic rice suspension cells. Transcript levels of the endogenous *αAmy3* and the transgene *mGM-CSF* were increased in the *OsMYBS2*-silenced suspension cells. The highest yield of recombinant mGM-CSF protein attained in the *OsMYBS2*-silenced transgenic suspension cells was 69.8 µg/mL, which is 2.5-fold that of non-silenced control cells. The yield of recombinant mGM-CSF was further increased to 118.8 µg/mL in cultured cells derived from homozygous F_5_ seeds, which was 5.1 times higher than that of the control suspension cell line.

**Conclusions:**

Our results demonstrate that knockdown of the transcription factor gene *OsMYBS2* increased the activity of the *αAmy3* promoter and improved the yield of recombinant proteins secreted in rice cell suspension cultures.

**Supplementary Information:**

The online version contains supplementary material available at 10.1186/s13007-021-00799-2.

## Background

Plant molecular farming is a technology used in genetic engineering whereby plants are used to produce valuable therapeutic recombinant proteins and secondary metabolites by transferring recombinant gene(s) to plant hosts [1]. With clear advantages in terms of biosafety and the cost of large-scale production, plant molecular farming has received attention as a powerful means of expressing recombinant proteins to yield pharmaceutical products, such as antibodies, enzymes, vaccines, and cytokines [2–4]. However, the application of transgenic plants in the field has raised concerns associated with subsequent purification, contamination of transgenes in the food chain via cross pollination, and strict government regulation of genetically modified crops. Transgenic plant suspension cells are cultured in a controlled sterile environment and can be upscaled using bioreactors, thus showing economic potential for the production of valuable bioproducts [5, 6].

For the production of recombinant proteins, the host cells of rice and tobacco are those used most frequently in plant suspension cultures. The best-known system of transgenic rice cell suspension culture is based on the rice *ALPHA-AMYLASE 3* gene (*αAmy3*, also termed *RAmy3D*) promoter, which is induced strongly by sugar starvation [7]. The signal peptide of *α*Amy3 allows recombinant proteins to be secreted into the liquid medium, thereby avoiding cell lysis and the complicated steps required for protein purification [8]. Several recombinant proteins have been produced using the *αAmy3* promoter and signal peptide in cultured cells of transgenic rice cell suspensions [9–16]. Although the *αAmy3* promoter has been used widely for sugar-regulated recombinant protein production [9], rice cells have been genetically engineered to improve the *αAmy3* promoter based-recombinant production system. For example, knockdown of endogenous *αAmy3* expression increased recombinant human GM-CSF production 1.9-fold in transgenic rice cells [17]; silencing of the expression of the *CYSTEINE PROTEASE* gene in transgenic rice cells resulted in an increase in the yield of recombinant human GM-CSF [18].

Sugar signals mediate transcriptional regulation of *αAmy3* [7]. A duplicate TA box in the *αAmy3* promoter is an essential regulatory motif for potent activation of the *αAmy3* promoter in sugar-starved rice cells [7, 19, 20]. The TA box can be bound by three sugar-repressible 1R-MYB transcription factors: OsMYBS1, OsMYBS2, and OsMYBS3 [21, 22]. Regulation of the promoter activity of *αAmy3* is achieved by competitive binding between OsMYBS1 and OsMYBS2 to the TA box of the *αAmy3* promoter [22]. OsMYBS1 activates the TA box-containing promoter [21, 23], whereas OsMYBS2 reduces promoter activity in rice cells under sugar depletion [21, 22]. Based on the study of overexpression and underexpression of *OsMYBS2* in transgenic rice cells, a lower level of OsMYBS2 is essential for potent activation of the *αAmy3* promoter under sugar depletion [22].

Granulocyte–macrophage colony-stimulating factor (GM-CSF) is an immune-response cytokine generated by macrophages, endothelial cells, and immune-stimulated fibroblasts [24–28]. The GM-CSF protein functions in the development and activation of myeloid precursor cells, macrophages, granulocytes, and dendritic cells [29–32]. GM-CSF has been used in various clinical applications, including as a vaccine adjuvant, in cancer therapy, and immunotherapy for malignancies [33–37]. GM-CSF shows species specificity; although it shares 54% amino acid sequence identity with human GM-CSF, mouse GM-CSF (mGM-CSF) is used preferentially in immune system- and cancer-related research.

In a previous study, we produced mGM-CSF using *αAmy3* promoter-based transgenic rice suspension cells with mGM-CSF demonstrated to accumulate to a maximum yield of 24.6 mg/L attained in a 2 L bioreactor [38]. The transcription factor OsMYBS2 reduces activation of the *αAmy3* promoter by competing with OsMYBS1 for binding to the TA box of the *αAmy3* promoter [22]. Therefore, the culture of genetically engineered rice suspension cells where OsMYBS2 activity is repressed is one potential strategy to increase production of a recombinant protein based on a cell suspension culture system. To evaluate the effectiveness of this strategy, we compared the yield of the recombinant protein mGM-CSF between wild-type (WT) and OsMYBS2-knockdown rice cell suspensions. The yield of recombinant mGM-CSF production increased to 118.8 µg/mL in OsMYBS2-knockdown cells compared with that seen in WT rice suspension cells. The present results demonstrate that production of the recombinant protein mGM-CSF can be enhanced using OsMYBS2-knockdown transgenic rice cell suspensions.

## Materials and methods

### Plant materials

**Fig. 1:**
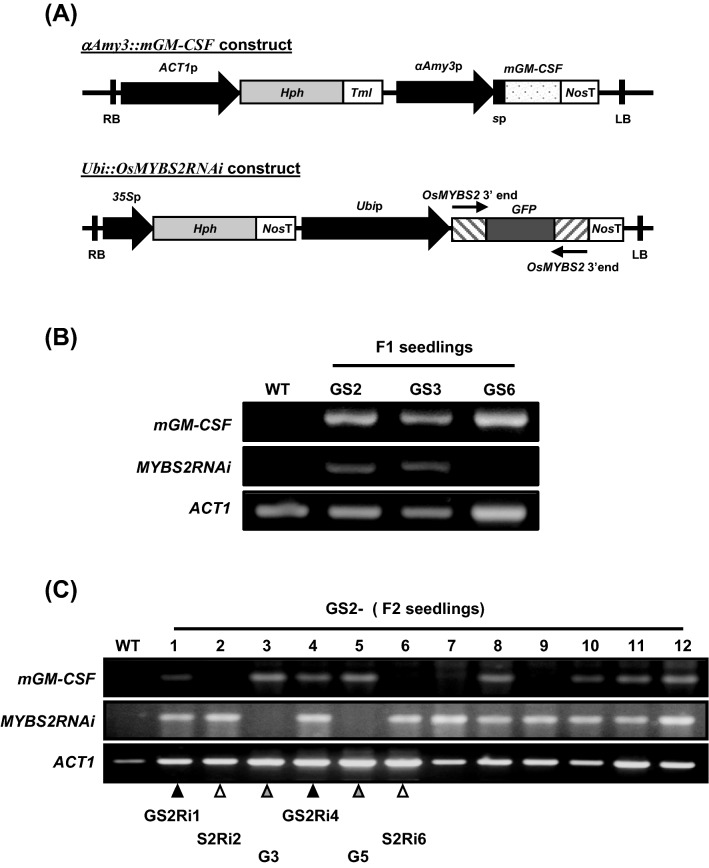
Generation of transgenic rice plants harboring the *αAmy3p::mGM-CSF* and *Ubip::OsMYBS2RNAi* chimeric genes. **A** Schematic representation of the expression cassettes in transgenic rice plants used for a dihybrid cross. For *αAmy3p::mGM-CSF*, the *mGM-CSF* cDNA was inserted downstream of the *αAmy3* promoter (*αAmy3*p)–signal peptide (sp) sequence. For *Ubip::OsMYBS2RNAi*, a 271 bp fragment at the 3′ end of the *OsMYBS2* cDNA was ligated in sense and antisense orientations to the *GFP* cDNA and fused downstream of the *Ubi* promoter (*Ubi*p). **B** and **C** PCR-based genotype detection of the *αAmy3p::mGM-CSF* and *Ubip::OsMYBS2RNAi* chimeric genes in F_1_ progeny (**B**) and F_2_ population derived from self-pollinated F_1_ plants (**C**). The primer sets for *mGM-CSF* and *OsMYBS2RNAi* were used to amplify specifically the *αAmy3p::mGM-CSF* and *Ubip::OsMYBS2RNAi* chimeric genes, respectively. Arrowheads indicate the lines GS2Ri1 and GS2Ri4 (*OsMYBS2* knockdown and *mGM-CSF* expression), S2Ri2 and S2Ri6 (*OsMYBS2* knockdown only), and G3 and G10 (*mGM-CSF* expression only), which were selected for further studies

Transgenic lines harboring the *αAmy3p::mGM-CSF* [38] and *Ubip::OsMYBS2RNAi* [22] transgenes were used in this study. The transgenic lines were in the ‘Tainung 67’ (TNG67) background and were generated by *Agrobacterium*-mediated transformation [22, 38]. The *αAmy3p::mGM-CSF* transgene contains a rice sugar depletion-inducible promoter, *αAmy3*p, and an αAmy3 signal peptide DNA fused upstream of the *mGM-CSF* gene (Fig. 1A). Recombinant mGM-CSF proteins were sucessfully produced in the *αAmy3* promoter-based transgenic rice suspension cells [38]. The *Ubip::OsMYBS2RNAi* transgene contains an *OsMYBS2* RNAi DNA fragment, which is an inverted repeat of the 271-base pair (bp) region at the 3ʹ untranslated region of *OsMYBS2* cDNA fused at the up- and downstream ends of a *GFP* coding sequence, under the control of the maize ubiquitin gene (*Ubi*) promoter (Fig. 1A). Knockdown of *OsMYBS2* expression in the *Ubip::OsMYBS2RNAi* transgenic lines has been reported previously [22].

To obtain single-copy transgene lines, these transgenic lines were selected based on 3:1 ratio of transgene from T_1_ generation for possible transformation events that the transgene was inserted at single locus in the genome. Genotype and expression levels of transgene were monitored from T_2_ to T_5_ generation of the transgenic lines. The *αAmy3p::mGM-CSF* and *Ubip::OsMYBS2RNAi* stable homozygous transgenic lines were obtained, respectively.

### Crossing

The *αAmy3p::mGM-CSF* homozygous transgenic line was used as the female parent and the *Ubip::OsMYBS2RNAi* homozygous transgenic line was used as the male parent. A panicle with spikelets from one of the *αAmy3p::mGM-CSF* homozygous transgenic lines was selected to cross. The top one third of each spikelet was cut off using fine pointed sharp scissors. Then the whole panicle was soaked in warm water at 43 ℃ for 5 min to inactivate the pollen harboured by the ather pollen sacs. Stamens were removed gently with fine-tip forceps. Pollination was performed with the pollen that had been sampled from the *Ubip::OsMYBS2RNAi* homozygous transgenic line to get the F1 hybrid seeds. Progenies of the F_2_, F_3_, F_4_, and F_5_ generations were raised by self-pollination.

### Establishment of rice cell suspension cultures

Seeds of the F_3_ and F_5_ progenies were dehulled, sterilized with 3% Sodium hypochlorite for 30 min, washed extensively with sterile water, placed on Murashige and Skoog (MS) [39] solid medium supplemented with 10 µM 2 4-dinitrophenylhydrazine (2,4-D) and 3% sucrose, and incubated at 28℃ in the dark. After 1 month, calli were transferred to 25 mL N6 [40] liquid medium containing 10 µM 2,4-D and 3% sucrose in a 250 mL flask and maintained on an orbital shaker at 110 rpm at 28℃ in a dark culture room. Suspension cells were subcultured every 7 days in fresh N6 liquid medium containing sucrose and 2,4-D.

### PCR-base genotype analysis

Genomic DNA was isolated from 2-week-old seedlings or calli [41]. The rice samples were ground by mortar and pestle with liquid nitrogen. The ground sample powder was transferred into a pre-chilled Eppendorf tube. Then, 0.75 mL extraction buffer (100 mM Tris–HCl pH 8.0, 50 mM EDTA pH 8.0, 100 mM NaCl, 1% SDS, 1% β-mercaptoethanol) was added to dissolve the sample powder, and all samples were incubated at 65℃for 15 min. The sample was mixed with 0.25 mL 3 M potassium acetate and incubated at -20℃ for 20 min. Then the sample solution was centrifuged at 10,000×*g* for 20 min, and post centrifuging, the sample supernatant was collected. One-fifth the volume of isopropanol was added to the supernatant for DNA precipitation. The sample was then centrifuged at 10,000×*g* for 20 min to pellet the DNA. After air-drying, the DNA pellet was dissolved in 50 µL ddH_2_O and preserved at − 20 ℃. One microgram of each genomic DNA sample was subjected to PCR using the following gene-specific primers: for amplification of *mGM-CSF* DNA, 5′-ATGGCACCCACCCGCTCACC-3′ and 5′-AAAAAACCAGTCCAAAAATGA-3′; for amplification of *OsMYBS2RNAi* DNA, 5′-AAAGGATCCCTCGAGATGGTGAGCAAGGGCGAG-3′ and 5′-GGGATCCTATCTAGACTTGTACAGCTCGTCCAT-3′; and for amplification of *ACTIN-1* (*ACT1*) DNA, 5′-CTGATGGACAGGTTATCACC-3′ and 5′-CAGGTAGCAATAGGTATTACAG-3′. The PCR products were separated by electrophoresis in 0.5 X TAE buffere at 100 V.

### Quantitative real-time polymerase chain reaction (qRT-PCR) analysis

Total RNA was isolated from caill as described previously [38]. The rice cell sample was ground by mortar and pestle with liquid nitrogen. The ground sample powder was transferred into a pre-chilled Eppendorf tube, and then 0.5 mL TRIzol Reagent (Invitrogen, Carlsbad, CA, USA) was added to the sample powder and incubated for 10 min at room temperature. The rice cell sample was mixed with 100 μL chloroform and incubated for 10 min at room temperature. The sample was centrifuged at 10,000×*g* for 20 min at 4 ℃ to collect the supernatant. Next, 250 μL isopropanol was added to the supernatant to precipitate the total RNA. After 10 min of incubation at room temperature, the sample was centrifuged at 10,000×*g* for 20 min at 4 ℃ to pellet the total RNA. The RNA pellet was washed with 500 μL of 75% ethanol. After removing the 75% ethanol wash, the RNA pellet was dried at room temperature for 10 min. The RNA pellet was than dissolved in 50 µL DEPC-treated water. Isolated total RNA was treated with RNase-free DNase I (NEB, Ipswich, MA, USA) to remove possible DNA contamination. First-strand complementary DNA (cDNA) was synthesized from 2.5 µg total RNA using ReverTra Ace® reverse transcriptase (Toyobo, Osaka, Japan) with oligo-dT primers. A tenfold dilution of the resultant first-strand cDNA was subjected to qRT-PCR with *mGM-CSF*, *OsMYBS2*, and *αAmy3* gene-specific primers using the FastStart Essential DNA Green Master (Roche, Basel, Switzerland) and the PikoReal™ Real-Time PCR system (Thermo, Waltham, MA, USA). For detection of *mGM-CSF* mRNA, the aforementioned specific primers for amplification of mGM-CSF DNA were used. The primers 5′-GGACTCGAGAATGGAGCAGCATGAGGA-3′ and 5′-GTCCATGGTACCCCTTTCTT-3′ were used for detection of *OsMYBS2* mRNA, and the primers 5′-GTAGGCAGGCTCTCTAGCCTCTAGG-3′ and 5′-AACCTGACATTATATATTGCACC-3′ were used to detect *αAmy3* mRNA. The qPCR procedure was repeated independently at least three times. Expression of *ACTIN-1* in rice suspension cells is repressed in sugar free medium, so 18S rRNA was applied as a reference gene for normalization in detection of gene expression under sugar starvation treatments. The primers used to detect 18S rRNA were 5′-CCTATCAACTTTCGATGGTAGGATA-3′ and 5′-CGTTAAGGGATTTAGATTGTACTCATT-3′. The relative gene expression level was expressed as the ratio of the target gene mRNA abundance to the 18S rRNA abundance. Data were analyzed using PikoReal 2.0 software (Thermo).

### Western blot analysis

Protein gel blot analysis was performed as described previously [38] and the concentration of the total protein of each sample was determined by using the Bio-Rad Protein Assay reagent (Bio-Rad, Hercules, CA, USA). The proteins were separated by 15% SDS-PAGE, and 20 µg of protein was loaded per lane. The separated proteins were then transferred onto a PVDF membrane. The polyclonal rabbit anti-mGM-CSF antibody (Abcam, Cambridge, MA, USA) was used as the primary antibody, and HRP-conjugated anti-rabbit IgG was used as the secondary antibody to detect rmGM-CSF protein. The signal was detected by chemiluminescence using ECL prime western blot detection (GE Healthcare, Chicago, IL, USA).

### Enzyme-linked immunosorbent assay (ELISA)

The concentration of rmGM-CSF in the culture medium was determined by sandwich ELISA following the method described by Liu et al. [38]. The goat anti-mGM-CSF polyclonal antibodies were coated onto 96-well microtiter plates, and then 50 µL of cell cultured medium protein samples were added into individual wells of a microtiter plate for 60 min incubation at 37 ℃. The rabbit anti-mGM-CSF polyclonal antibodies (Abcam) were added to the wells and incubated at 37 ℃ for 60 min. Goat peroxidase-conjugated anti-rabbit IgG antibodies applied for the detection of rabbit IgG antibody were added and incubated at 37 ℃ for 60 min. A substrate of ABTS solution (Sigma, St Louis, MO, USA) was added to the wells. The optical density at 450 nm of each well was recorded using an Epoch Multi-Volume Spectrophotometer System (BioTek, Winooski, VT, USA).

## Results and discussion

### Generation of *OsMYBS2* knockdown and *mGM-CSF* expressing rice plants by a dihybrid cross

Variability of transgene expression is frequently observed in independent transgenic lines due to several factors, such as differences in chromosomal position and transgene copy number. To eliminate genetic background effects on the yield of recombinant mGM-CSF protein between WT and *OsMYBS2*-knockdown rice suspension cells, a dihybrid cross approach was used to generate the *OsMYBS2*-knockdown and *mGM-CSF*-expressing transgenic rice plants. The homozygous transgenic rice harboring the *αAmy3p::mGM-CSF* transgene (Fig. 1A) was used as the female plant, which was crossed with the *Ubip::OsMYBS2RNAi* (Fig. 1A) homozygous transgenic plant line which was used as the male plant. Given that both parental transgenic rice lines were generated under the genetic background of the rice cultivar Tainung 67 (TNG67) by *Agrobacterium*-mediated transformation, the progenies derived from the dihybrid crossing event were assumed to have only difference of the transgene copy numbers. Three F_1_ seeds were obtained and the genotype of the F_1_ seedlings was analyzed using PCR-based genotype detection. Two F_1_ offspring, GS2 and GS3, were dihybrid heterozygous for the *OsMYBS2RNAi* and *mGM-CSF* transgenes (Fig. 1B). Subsequently, the F_2_ progeny were obtained from self-pollination of the GS2 individual. Genotype analysis revealed that four F_2_ progeny carried the *OsMYBS2RNAi* gene, namely plants GS2-2, -6,-7, and -9, and two F2 progeny were determined to harbor the mGM-CSF transgene, namely plants GS2-3 and -5. In addition, PCR-based genotyping revealed that six F2 progeny harbored by the mGM-CSF and OsMYBS2RNAi transgenes, including plants GS2-1, -4, -8, -10, -11, and -12 (Fig. 1C). For further investigation, GS2-2 and GS2-6 contained the transgene *OsMYBS2RNAi* and were renamed as S2Ri2 and S2Ri6. Next, GS2-3 and GS2-5 carried the transgene *mGM-CSF* and were renamed as G3 and G5. Finally, GS2-1 and GS2-4 had both transgenes and were rename as GS2Ri1 and GS2Ri4 (Fig. 1C).

### Establishment of *OsMYBS2*-knockdown and *mGM-CSF*-expressing transgenic rice suspension cells

**Fig. 2:**
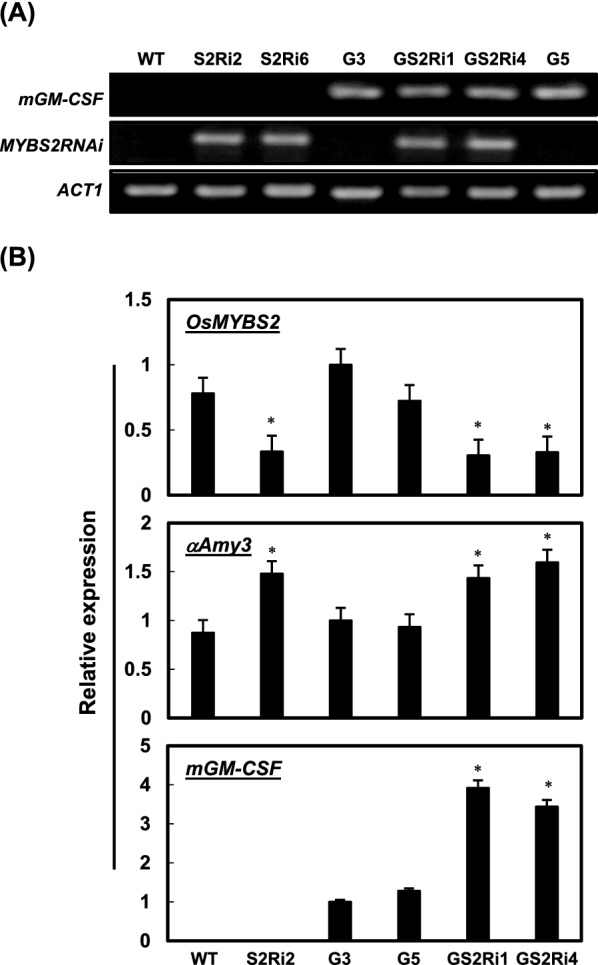
Establishment and characterization of *mGM-CSF/OsMYBS2RNAi* transgenic rice suspension cell lines. **A** Genotyping of suspension cell lines. PCR-based detection of *αAmy3p::mGM-CSF* and *Ubip::OsMYBS2RNAi* chimeric genes was conducted on various calli derived from F_3_ seeds. The primer sets for *mGM-CSF* and *OsMYBS2RNAi* were used to amplify specifically the *αAmy3p::mGM-CSF* and *Ubip::OsMYBS2RNAi* chimeric genes, respectively. S2Ri2 and S2Ri6 were *OsMYBS2* knockdown lines, G3 and G5 were *mGM-CSF* transgenic lines, and GS2Ri1 and GS2Ri4 were *mGM-CSF/OsMYBS2RNAi* lines. **B** Expression of *OsMYBS2*, *αAmy3*, and *mGM-CSF* in the established rice suspension cell lines. Total RNA was isolated from suspension cells sugar-starved for 2 days and then subjected to qRT-PCR using primers specific for *OsMYBS2*, *αAmy3*, and *mGM-CSF*. Error bars indicate the standard deviation (SD) of triplicate experiments. For *OsMYBS2* and *αAmy3*, gene expression was relative to that of wild-type (WT) suspension cells, where 1 = equivalent. For *mGM-CSF*, *g*ene expression was relative to that of G3 suspension cells, where 1 = equivalent. * Significantly different from the control cell lines (Student’s *t*-test: *p* < 0.05)

To investigate the effect of *OsMYBS2* knockdown on *αAmy3p::mGM-CSF* transgene expression, calli were induced from F_3_ seeds of the WT, two *OsMYBS2RNAi* only lines, S2Ri2 and S2Ri6, two *mGM-CSF* only lines, G3 and G5, and two *mGM-CSF*/*OsMYBS2RNAi* lines, GS2Ri1 and GS2Ri4. Given that the parental rice lines were generated in the TNG67 background by *Agrobacterium*-mediated transformation, the progeny of this dihybrid cross were genetically identical, except for the composition of the sequences of the two introduced transgenes. Chen et al. [22] has reported that OsMYBS2 is a negative regulator of *αAmy*3 expression. Transgenic rice plants constitutively overexpressing *OsMYBS2* exhibited reduced seed germination, delayed seedling growth, and shorted mature plant height, while knockdown of *OsMYBS2* expression did not show any obvious phenotypes [22]. In this study, callus induction rates from these knockdown transgenic lines were determined to be similar to those of wild-type rice plants. After genotyping of progeny plants (Fig. 2A), calli of these transgenic lines were used to establish cell suspension cultures which exhibited similar cell morphology to WT (Additional file 1: Fig. S1). Subsequently, *mGM-CSF* mRNA levels were compared in these cell suspension lines under sugar starvation. In addition to the WT control cell line, one of *OsMYBS2RNAi* only cell lines, S2Ri2, was selected as another control for the knockdown genetic background of *OsMYBS2*. Total RNA was isolated from the WT, S2Ri2, G3, G5, GS2Ri1, and GS2Ri4 cell suspension lines sugar-starved for 48 h and subjected to qRT-PCR analysis. Expression of *OsMYBS2* was lower in the S2Ri2, GS2Ri1, and GS2Ri4 lines than in the WT, G3, and G5 cell lines, as expected (Fig. 2B). The expression of *mGM-CSF* transgene was controlled by the sugar-starvation-inducible *αAmy3* promoter, so mRNA of the transgene was detected only in the sugar-starved G3, G5, GS2Ri1, and GS2Ri4 cell lines (Fig. 2B). Comparison of the various cell lines revealed that *mGM-CSF* mRNA levels were significantly higher in both *mGM-CSF/OsMYBS2RNAi* lines, GS2Ri1 and GS2Ri4, than in the G3 and G5 cell lines (Fig. 2B). Similarly, the *αAmy3* mRNA levels were higher in the S2Ri2, GS2Ri1, and GS2Ri4 cell lines than in the WT, G3, or G5 cell lines (Fig. 2B). These results indicate that knockdown of *OsMYBS2* increased the expression of the *αAmy3p::mGM-CSF* transgene in sugar-starved cells.

### Knockdown of *OsMYBS2* expression increases production of recombinant mGM-CSF protein in the culture medium

**Fig. 3:**
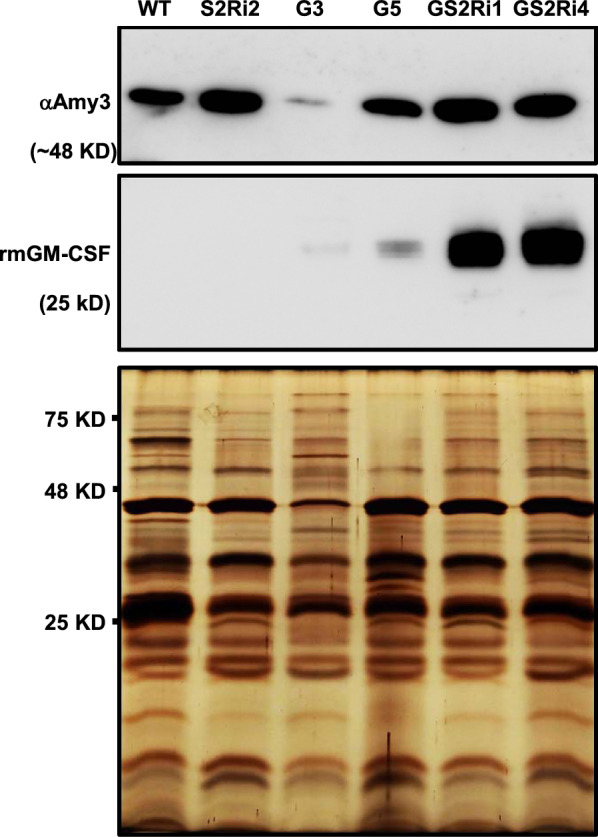
Production of recombinant mGM-CSF in the culture medium of *mGM-CSF/OsMYBS2RNAi* transgenic rice suspension cell lines. One milliliter of suspension cells, consisting of the wild type (WT), one *OsMYBS2* knockdown line S2Ri2, two *mGM-CSF* gene transgenic lines G3 and G5, and two *OsMYBS2* knockdown and *mGM-CSF* transgenic lines GS2Ri1 and GS2Ri4, were cultured in 2 mL sugar-free N6 medium for 5 days. Samples of the culture medium were collected to determine *α*Amy3 and rmGM-CSF abundance by western blot analysis with specific antibodies to *α*Amy3 and mGM-CSF, respectively. Silver staining was used to and visualize bands in the culture medium and represent as the loading control

To examine whether knockdown of *OsMYBS2* expression enhanced production of the recombinant mGM-CSF (rmGM-CSF) protein, the rmGM-CSF protein productivity was compared among two *mGM-CSF* control cell lines, G3 and G5, and two *mGM-CSF/OsMYBS2-RNAi* cell lines, GS2Ri1 and GS2Ri4. Cell suspensions were cultured in sugar-containing N6 medium for 3 days, and then 1.0 mL of cultured cells was incubated in 2.0 mL sugar-free N6 medium for 5 and 7 days. The cultured medium of each cell line was collected and an equal amount of total medium protein from each line was analyzed by western blot analysis. After sugar starvation for 5 days, the amount of recombinant mGM-CSF (rmGM-CSF) protein detected in the sugar-free liquid N6 medium of the GS2Ri1 and GS2Ri4 cell lines was considerably higher than that detected in the medium of the control cell lines G3 and G5 (Fig. 3). Higher amounts of *α*Amy3 protein were detected in the medium of the GS2Ri1 and GS2Ri4 cell lines than in that of the G3 and G5 cell lines (Fig. 3). Similar results for enhanced rmGM-CSF production in *OsMYBS2-*knockdown cell lines were obtained after sugar starvation for 7 days (Additional file 1: Fig. S2). These results indicate that knockdown of *OsMYBS2* expression improved rmGM-CSF production in the medium of sugar-starved rice cell suspension cultures.

The OsMYBS2 protein acts as a weak transcription activator that competes to bind to the TA box of the *αAmy3* promoter with the strong transcription activator, OsMYBS1, and leads to the low activity of the *αAmy3* promoter [21, 22]. In sugar-starved rice suspension cells, OsMYBS2 is relieved from the TA box, while SnRK1A regulates OsMYBS1 binding to the TA box to stimulate activation of the *αAmy3* promoter [22, 42]. The present results show that *mGM-CSF* mRNA levels and recombinant mGM-CSF protein were more abundant in the *mGM-CSF/OsMYBS2RNAi* suspension cell lines than in the *mGM-CSF* control cell lines under sugar starvation (Fig. 3). This finding indicates that *OsMYBS2* knockdown enhanced *αAmy3* promoter activity, and transcription of the *αAmy3p::mGM-CSF* transgene was increased, thereby imporoving recombinant mGM-CSF production from sugar-starved rice suspension cells.

### Profiling of rmGM-CSF production in the culture medium of *αAmy3p::mGM-CSF/ OsMYBS2RNAi* rice suspension cells

**Fig. 4:**
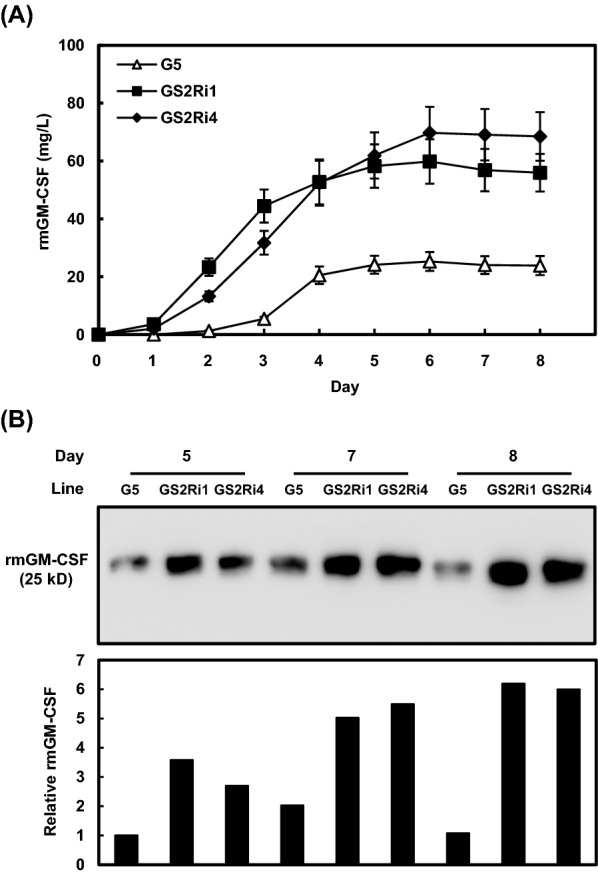
Recombinant mGM-CSF production profiling in the culture medium of *mGM-CSF/OsMYBS2RNAi* transgenic rice suspension cell lines. **A** One milliliter of suspension cells were cultured in 2 mL sugar-free N6 medium for various periods. Samples of the culture medium were collected to determine the concentration of rmGM-CSF by ELISA. Error bars represent the SD from triplicate cultures. **B** Sample of the culture medium collected at days 5, 7, and 8, and an equal amount of total medium protein from each sample were subjected to western blot analysis using mGM-CSF antibodies. The relative levels of rmGM-CSF abundance were measured using ImageJ software. The rmGM-CSF level was relative to that of G5 suspension cells at day 5, where 1 = equivalent

To determine the optimal period for rmGM-CSF production, rice suspension cells at an initial density of 50% (v/v) were cultured in sugar-free N6 medium for various periods and protein yields of rmGM-CSF were monitored using an enzyme-linked immunoassay (ELISA). Similar to a previous report [38], rmGM-CSF was detected initially from day 2, and the yield increased to a maximum of 25.28 mg/L on day 5 and was maintained until day 8 in the G5 control line (Fig. 4A). The rmGM-CSF protein was detected initially from day 1 in the culture medium of the GS2Ri1 and GS2Ri4 cell lines (Fig. 4A). The concentration of rmGM-CSF produced by the GS2Ri1 and GS2Ri4 cell lines increased rapidly and attained maximal concentrations of 59.84 and 69.77 mg/L, respectively, on day 6 to day 8 (Fig. 4A). Compared with the G5 control cell line, the GS2Ri1 and GS2Ri4 suspension cells produced 2.4–2.9 times higher amounts of rmGM-CSF in the sugar-containing culture medium, regardless of culture duration (4, 5, 6, 7, and 8 days) (Fig. 4A). To confirm this conclusion, the rmGM-CSF abundance was compared between the G5 and two *αAmy3p::mGM-CSF/OsMYBS2-RNAi* lines by western blot analysis with mGM-CSF antibodies. The relative abundance of rmGM-CSF was measured using ImageJ software. Regardless of the sugar-free culture medium from days 5, 7 and 8, the rmGM-CSF abundance in the GS2Ri1 and GS2Ri4 cell lines was at least twofold more abundant than it was in the G5 suspension cells (Fig. 4B). These results indicate that knockdown of *OsMYBS2* expression led not only to an increase in rmGM-CSF abundance but also to earlier production in the culture medium.

In the rice suspension culture recombinant protein expression system, cells are initially generally cultured in a sugar-containing medium for cell proliferation and growth. The recombinant protein is produced after cells are transferred to a sugar-free medium. However, when recombinant proteins are produced from sugar-starved cells, the cell viability decreases [12, 38, 43]. We observed that knockdown of *OsMYBS2* expression accelerated the accumulation of rmGM-CSF protein in the medium, which may be caused by rapid stimulation of *αAmy3* promoter activity in response to sugar depletion in the presence of reduced OsMYBS2 abundance. Acceleration of the *αAmy3* promoter response to sugar depletion is particularly advantageous for production of recombinant proteins by the *αAmy3* promoter-based recombinant protein expression system because it shortens the duration of recombinant protein production and reduces cell damage. In addition, recombinant protein production is maintained at relatively stable levels under repeated cycles of the presence and absence of sugar by the same batch of rice suspension cells [12, 43]. An early and rapid response to sugar depletion also provides the basis to accelerate the rmGM-CSF production cycle using a repeated-cycle culture strategy in bioreactors.

### Stable enhancement of rmGM-CSF production from *αAmy3p::mGM-CSF/OsMYBS2RNAi* rice suspension cells derived from F_3_ and F_5_ seeds

A dihybrid cross was used to produced trangenic rice plants containing both *αAmy3p::mGM-CSF* and *Ubip::OsMYBS2RNAi* transgenes. Based on genetic laws, F_1_ progeny should be heterozygous at both loci (*OsMYBS2RNAi* and *mGM-CSF* transgenes), so only one copy of each transgene existed in F_1_ seeds. After self-pollination of F_1_ plants, dihybrid homozygous transgenic rice plants that contain two copies of each transgenes, *OsMYBS2RNAi* and *mGM-CSF*, can be obtained in F_2_ population. Genotype analysis of progeny from self-pollinated F_2_ plants revealed that all 86 analyzed individuals from either GS2Ri1 or GS2Ri4 parents were *mGM-CSF* only or *mGM-CSF/OsMYBS2RNAi* transgenic lines (Additional file 1: Table S1). This result suggests that progeny in the F_3_ population was homozygous for the *mGM-CSF* transgene, but remained heterozygous for the *OsMYBS2RNAi* transgene. Genotype analysis of the F_4_ progeny showed that both *mGM-CSF* and *OsMYBS2RNAi* transgenes were homozygous in the GS2Ri1-2 and GS2Ri1-4 populations (Additional file 1: Table S1). Subsequently, progeny homozygous for *mGM-CSF* and *OsMYBS2RNAi* transgenes were obtained in the F_5_ populations, which were derived from self-pollination of GS2Ri1-2-1, GS2Ri4-1-2, and GS2Ri4-4-1 plants (Additional file 1: Table S1 and Fig. S3).

**Fig. 5:**
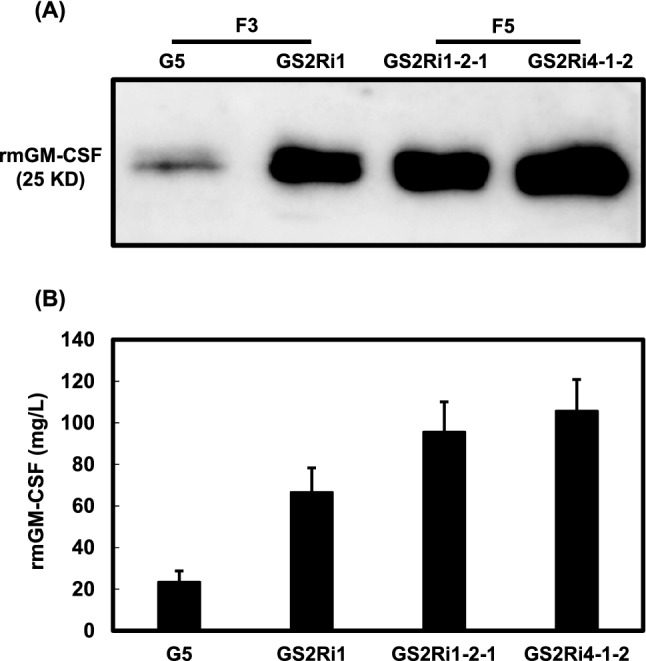
Production of rmGM-CSF in the F_5_ generation of *mGM-CSF/OsMYBS2RNAi* transgenic rice suspension cell lines. One milliliter of suspension cells, consisting of G5 (*mGM-CSF* only) and GS2Ri (*mGM-CSF/OsMYBS2RNAi*), both derived from F_3_ seeds, and GS2Ri1-2-1-1 and GS2Ri4-1-2-2 (both *mGM-CSF/OsMYBS2RNAi*), derived from F_5_ seeds, were cultured in 2 mL sugar-free N6 medium for 5 days. **A** Samples of culture medium were collected and equal amounts of total medium protein from each sample were subjected to western blot analysis using mGM-CSF antibodies. **B** The amount of rmGM-CSF was measured in the culture medium by ELISA. Error bars represent the SD from triplicate cultures

Gene dosage is correlated with expression level and, in the majority of cases, an increase in gene copy number enhances the expression of genes [44]. Suspension cell lines of GS2Ri1 and GS2Ri4 were derived from the F_2_ seed population, in which the *mGM-CSF* and *OsMYBS2RNAi* transgenes were either homozygous or heterozygous. Therefore, GS2Ri1 and GS2Ri4 cells must contain at least one copy of the *mGM-CSF* and *OsMYBS2RNAi* transgenes. To test whether increased dosage of the *mGM-CSF* and *OsMYBS2RNAi* transgenes in rice suspension cells can further increase production of rmGM-CSF, homozygous F_5_ seeds were used to establish suspension cell lines of GS2Ri1-2-1 and GS2Ri4-1-2, both containing two copies of the *mGM-CSF* and *OsMYBS2RNAi* transgenes. Western blot analysis and ELISA were used to compare the production of rmGM-CSF with the control cell line G5 and the F_3_ seed-derived cell line GS2Ri1. The concentration of rmGM-CSF in the culture medium of the GS2Ri1-2-1 and GS2Ri4-1-2 cell lines after sugar depletion for 5 days was 95.6 and 118.8 mg/L, which were 4 and 5.1 times higher than that of the G5 control cell line, respectively (Fig. 5). This result indicates that the yield of rmGM-CSF was further increased using F_5_ seed-derived suspension cell cultures.

Various strategies have been used to improve recombinant protein productivity in the rice suspension cell culture system based on the *αAmy3* promoter and signal peptide. Liu et al. [38, 43] used a one-step cultivation strategy in which the medium was not changed, but rather sucrose became depleted naturally, to improve recombinant protein production in rice suspension cell cultures. Knockdown of either *αAmy3* or *CysP* by RNAi increased the yield of human GM-CSF recombinant protein by approximately 2.5-fold. [17, 18]. The present study shows that rmGM-CSF production was enhanced by a factor of 5.1 using an *OsMYBS2* gene-silencing strategy. A strategy that combines all previous approaches is expected to further improve recombinant protein production in rice suspension cells. In addition to the *αAmy3* promoter, a modified rice *αAmy8* promoter has been used to produce a recombinant human epidermal growth factor in rice suspension cells and seedlings [45]. Knockdown of *OsMYBS2* expression increases the mRNA level of *αAmy8* in rice suspension cells [22], the promoter of which also contains a TA box [7]. Therefore, an increase in the production of recombinant protein derived from *αAmy8* promoter activity can be expected using an *OsMYBS2* gene-silencing strategy.

## Conclusions

The function of the rice transcription factor OsMYBS2 underlies the mechanism of sugar regulation of the *αAmy3* promoter. Reduction of *OsMYBS2* expression is essential for the strong activation of the *αAmy3* promoter in rice suspension cells under conditions of sugar depletion. On this basis, through knockdown of OsMYBS2 expression, *αAmy3* promoter activity can be increased, thereby facilitating and accelerating the accumulation of secreted recombinant proteins in rice cell suspension cultures.

## Supplementary Information

**Additional file 1: Table S1.** Genotyping result of seedlings. **Fig. S1.** Suspension rice cell morphology of the WT, two *OsMYBS2RNAi* only lines, S2Ri2 and S2Ri6, two *mGM-CSF* only lines, G3 and G5, and two *mGM-CSF*/*OsMYBS2RNAi* lines, GS2Ri1 and GS2Ri4. **Fig. S2.** Abundance of rmGM-CSF in *mGM-CSF/OsMYBS2RNAi* transgenic rice suspension cell lines was higher than that in mGM-CSF only transgenic lines. **Fig. S3.** PCR-based detection of *αAmy3::mGM-CSF* and *Ubi::OsMYBS2RNAi* chimeric genes in selected F5 progeny derived from self-pollination of the F4 population.

## Data Availability

All data generated or analyzed during this study are included in this published article and its supplementary imformation.

## Abbreviations

mGM-CSF: Mouse granulocyte–macrophage colony-stimulating factor
rmGM-CSF: Recombinant protein mGM-CSF
αAmy3: Alpha-amylase 3
WT: Wild type
*αAmy3*p: *αAmy3* Promoter
*Ubip*: Maize ubiquitin promoter
GFP: Green fluorescent protein
2,4-D: 2 4-Dinitrophenylhydrazine
PCR: Polymerase chain reaction
ACT1: Rice actin 1 gene
qRT-PCR: Quantitative real-time polymerase chain reaction
ELISA: Enzyme-linked immunosorbent assay
