# Supplementary material for: Knockdown expression of a MYB-related transcription factor gene, OsMYBS2, enhances production of recombinant proteins in rice suspension cells
Source: Plant Methods. 2021 Sep 25;17:99. doi: 10.1186/s13007-021-00799-2 (PMC8464127; doi:10.1186/s13007-021-00799-2)
Supplement: Supplementary file 1 — Additional file 1: Table S1. Genotyping result of seedlings. Fig. S1. Suspension rice cell morphology of the WT, two OsMYBS2RNAi only lines, S2Ri2 and S2Ri6, two mGM-CSF only lines, G3 and G5, and two mGM-CSF/OsMYBS2RNAi lines, GS2Ri1 and GS2Ri4. Fig. S2. Abundance of rmGM-CSF in mGM-CSF/OsMYBS2RNAi transgenic rice suspension cell lines was higher than that in mGM-CSF only transgenic lines. Fig. S3. PCR-based detection of αAmy3::mGM-CSF and Ubi::OsMYBS2RNAi chimeric genes in selected F5 progeny derived from self-pollination of the F4 population. [file 13007_2021_799_MOESM1_ESM.pdf]

Table S1. Genotype of seedlings from F1 to F5

Generation	Line name	Recombinant DNAs Containing				Total line
		rmGM-CSF only	OsMYBS2RNAi only	rmGM-CSF and OsMYBS2RNAi	wild type	
F1 seedlings	GS-	1	-	2	0	3
F2 seedlings	GS2Ri-	3	9	13	0	25
	GS3Ri-	4	3	11	0	18
F3 seedlings	GS2Ri1-	15	0	36	0	51
	GS2Ri4-	10	0	25	0	35
F4 seedlings	GS2Ri1-2-	0	0	10	0	10
	GS2Ri1-4-	0	0	9	0	9
	GS2Ri4-1-	2	0	6	0	8
	GS2Ri4-4-	5	0	5	0	10
F5 seedlings	GS2Ri1-2-1-	0	0	5	0	5
	GS2Ri4-1-2-	0	0	10	0	10
	GS2Ri4-4-1-	0	0	8	0	8

Supplementary Fig. 1

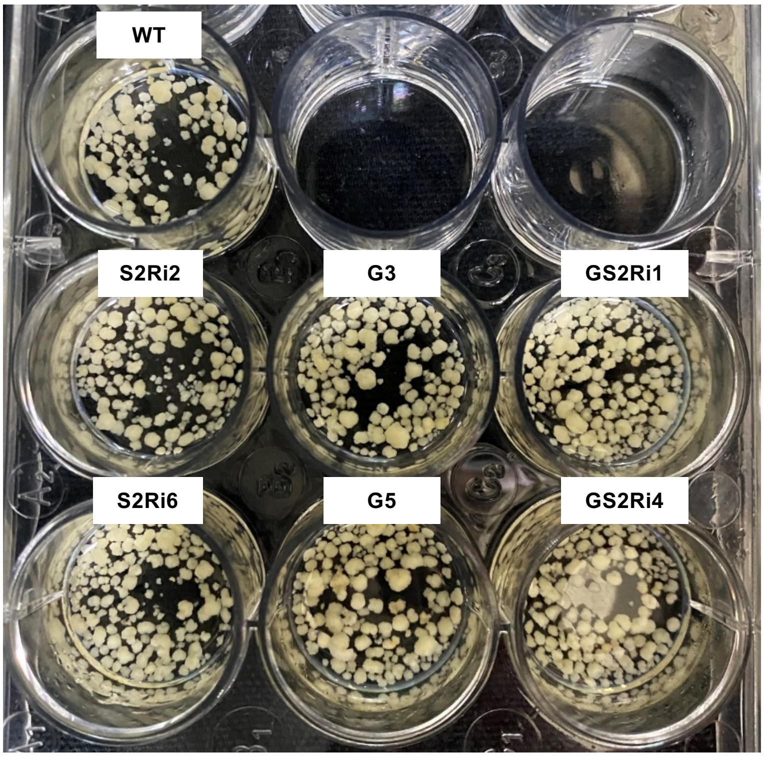

Fig. S1. Suspension rice cell morphology of the WT, two *OsMYBS2RNAi* only lines, S2Ri2 and S2Ri6, two *mGM-CSF* only lines, G3 and G5, and two *mGM-CSF*/*OsMYBS2RNAi* lines, GS2Ri1 and GS2Ri4. Photos were taken from suspension cultured rice cells plated in N6 liquid medium for 2 days.

Supplementary Fig. 2

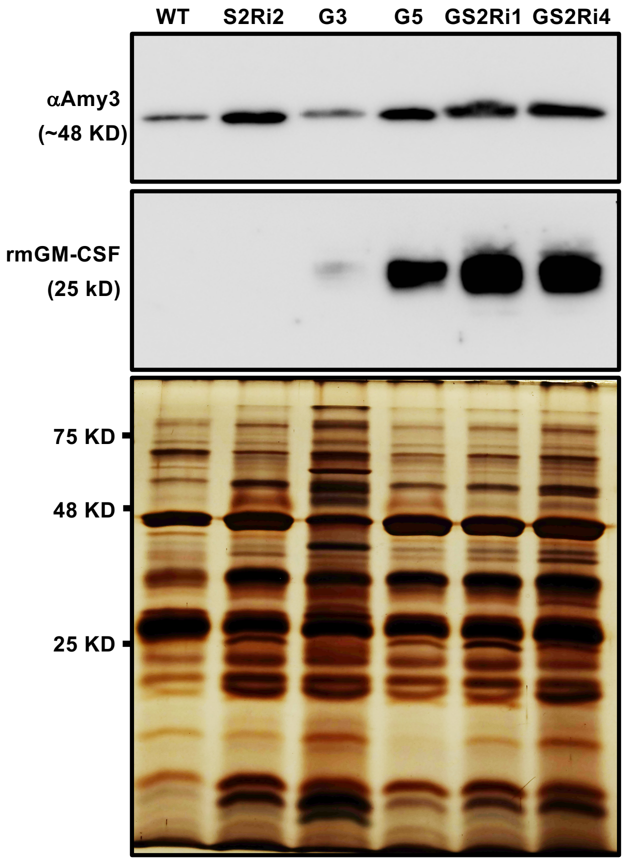

Fig. S2. Abundance of rmGM-CSF in *mGM-CSF/OsMYBS2RNAi* transgenic rice suspension cell lines was higher than that in mGM-CSF only transgenic lines.

One milliliter of suspension cells, consisting of the wild type (WT), one OsMYBS2 knockdown line S2Ri2, two mGM-CSF gene transgenic lines G3 and G5, and two OsMYBS2 knockdown and mGM-CSF transgenic lines GS2Ri1 and GS2Ri4, were cultured in 2 mL sugar-free N6 medium for 7 days. Samples of culture medium were collected to determine αAmy3 and rmGM-CSF by western blot analysis with specific antibodies to αAmy3 and mGM-CSF, respectively. Silver staining was used to visualize bands in the culture medium and represent the loading control.

Supplementary Fig. 3

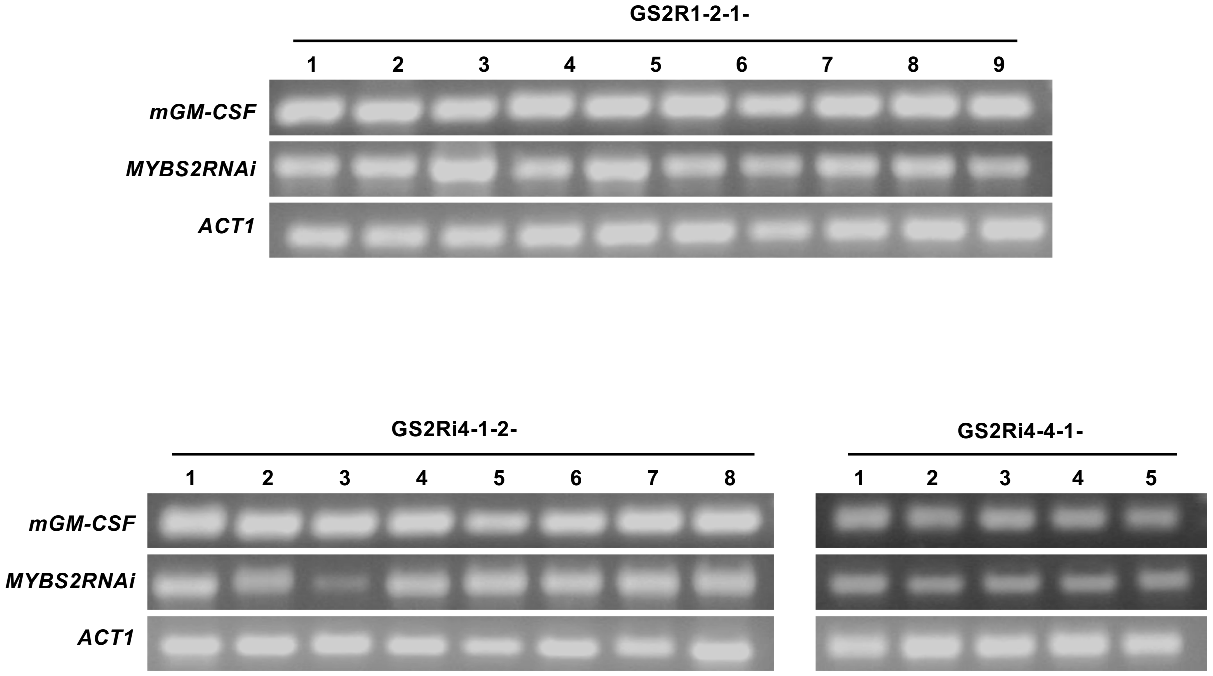

Fig. S3. PCR-based detection of *αAmy3::mGM-CSF* and *Ubi::OsMYBS2RNAi* chimeric genes in selected F5 progeny derived from self-pollination of the F4 population. The primer sets for *mGM-CSF* and *MYBS2RNAi* were used to amplify specifically the *αAmy3::mGM-CSF* and *Ubi::OsMYBS2RNAi* chimeric genes, respectively.
